# Optimizing cold storage for uniform airflow and temperature distribution in apple preservation using CFD simulation

**DOI:** 10.1038/s41598-024-76385-y

**Published:** 2024-10-25

**Authors:** Leo Daniel Alexander, Sanjeev Jakhar, Mani Sankar Dasgupta

**Affiliations:** 1 Smart Building Laboratory, Department of Mechanical Engineering, Birla Institute of Technology & Science, Pilani, Rajasthan 333031 India; 2 School of Mechanical Engineering, Vellore Institute of Technology, Chennai campus, Chennai, Tamil Nadu 600127 India

**Keywords:** Computational fluid dynamics, Cold storage, Porous medium, Post harvest, Air flow distribution, Temperature heterogeneity., Mechanical engineering, Renewable energy

## Abstract

Apples are preserved in cold storage within standard size crates to avoid injury during handling and are stacked in a specific manner to promote adequate air circulation. This research builds an air flow and heat transfer model of a cold room (5.75 m × 3.83 m × 3.75 m) with apple filled crates (0.55 m × 0.37 m × 0.3 m) modeled as a porous media and uses CFD simulation to study how alternate stacking impacts airflow distribution and product temperature. The conventional arrangement of crates, termed CS1, was simulated, and the resulting temperature distribution data were used to validate the model with published experimental data, a root mean square error of 1.13 °C indicates good match. The model is extended to examine temperature distribution for two additional arrangements of crates (CS2 and CS3) with changed orientations and spacing, in accordance with a specific strategy. CS3, featuring larger spacing along the z-direction, showed higher average air velocity compared to CS2 and CS1 by 7.4% and 3.7% respectively. CS3 also improved cooling rate by 25.2% and increased the number of chilled crates by 20% within 40 h, along with a reduced temperature heterogeneity (3.59 °C). The model could predict hot spots in various stacking configurations, aiding in optimal arrangement.

**Supplementary Information:**

The online version contains supplementary material available at 10.1038/s41598-024-76385-y.

## Introduction

Precooling is a process in which field heat is rapidly removed from freshly harvested produce, thereby reducing its perspiration rate to reduce moisture loss and extend the postharvest life^1^. Forced convective cooling process is one of the widely used methods of cooling inside a cold storage^2^. During this process, cold storage is maintained within a narrow range of optimum storage conditions and for apples, the same is 2 to 4 °C and at 75 to 95% relative humidity^3^. Apples are generally stored in standard size high density polyethylene (HDPE), polypropylene (PP) or wooden crates that are stacked on top of and alongside each other directly or supported on racks in a certain fashion to promote adequate air circulation^4^. The primary objective of using crates is to facilitate handling, avoid injury to the fruit and allow adequate cold air circulation for rapid cooling^5^. Due to their location in cold storage, some crates may experience a higher cooling rate than others. The cooling rate in cold storage is dependent on the type of packaging, the produce respiration rate, infiltration heat load, air circulation inside the cold storage and stacking of the crates^6^. Improper air circulation can create hot spots at various locations inside the cold storage resulting in a heterogeneous temperature distribution. Higher than optimal storage temperature induces a higher respiration rate as well as greater transpiration while lower than optimal storage temperature may cause chilling injury to the produce surface^7^. In addition to suboptimal operation, conventional refrigeration systems in cold storages contribute to increased energy consumption, adversely impacting the environment by contributing to global warming and ozone depletion^8^.

The simulation of real-life fruits in storage, including their geometrical and surface properties, packing factor, effect of fluid flow around them, and interaction of heat between the fruit and the circulating air, is a highly arduous task. Multiscale simulation is a relatively new implementation in the field of CFD, and its usage has been extended in the simulation of cold storage^9^. Multiscale simulation has been reported to be used to study the effects of several phenomena such as high-pressure fogging systems for pest control around packed storages, stacking patterns within a cold storage and evaporator dynamics, and the results agree well with the experimental data^10^. The research indicates that multiscale simulations not only simplify the simulation of real-life fruits in cold storage but also contribute to a reduction in computation time. Various researchers have reported multiple CFD simulations with different turbulence models to identify the most suitable model for predicting the temperature and air flow distribution in cold storage^11^. The results showed that all turbulence models predict the temperature and air flow distribution within a narrow range, deviating from the actual values, except for in the low Reynolds number region where the SST $\:k-\omega\:$ model predictions are significantly better. CFD simulations of cold storage have reported that products stored in crates stacked at the bottom tend to have higher chilling injury than those stored on the top because of the nature of the air flow distribution inside cold storage^12^. The effects of geometry and ventilation hole size on horticultural products were explored. The conclusion drawn was that packaging with larger volumes and larger ventilations demonstrated superior cooling capabilities^13^. In another study, a transient 3D CFD was employed to predict the air flow patterns and temperature distribution within three novel packaging box designs for storing horticultural products, and the authors reported that the proposed designs achieved temperatures 1.5 °C to 5 °C lower than those of conventional designs^14^. The influence of the size and placement of vent holes was studied, and it was reported that packaging boxes with fewer, larger, and strategically distributed vent holes improve the temperature homogeneity inside the box^15^. A transient 3D CFD model was also employed to study the effect of lateral and vertical stacking on the air flow and heat transfer through convection and conduction within the fruit^16^. In one other CFD study, an “entrainment effect” was reported in which some amount of warm return air was entrained and recirculated into the room without entering the IDU (Indoor unit), which is the cooling unit for the cold storage, causing unfavorable heating of the crates near the IDU^17^. Although many experimental and numerical investigations have been reported previously to model and comprehend the behavior of the air flow distribution and temperature field in cold storage^1^, the influence of stacking orientation and different crate spacings has not yet been adequately explored.

The objective of this research was to determine whether various stacking arrangements inside cold storages have significant effects on the crate temperature (average temperature of apples inside a single crate), number of cooled crates, air flow distribution and temperature heterogeneity inside cold storage. The simulation outcomes are compared and constructed against published experimental results of the same similitude to validate the model. The validated model is then extended to study the effect of changes in various cold storage configurations, such as stacking orientation and inter-crate spacing, in terms of identified measurable parameters, such as the crate temperature and air flow distribution. Finally, an optimized stacking configuration is selected from the proposed configurations based on having the lowest crate temperature and the least temperature heterogeneity compared to all other studied configurations. This study provides valuable insight into the temporal and spatial variations in cold storage temperature under various stacking arrangements and indicates the scope of optimization.

## Methods

### Computational model

**Fig. 1:**
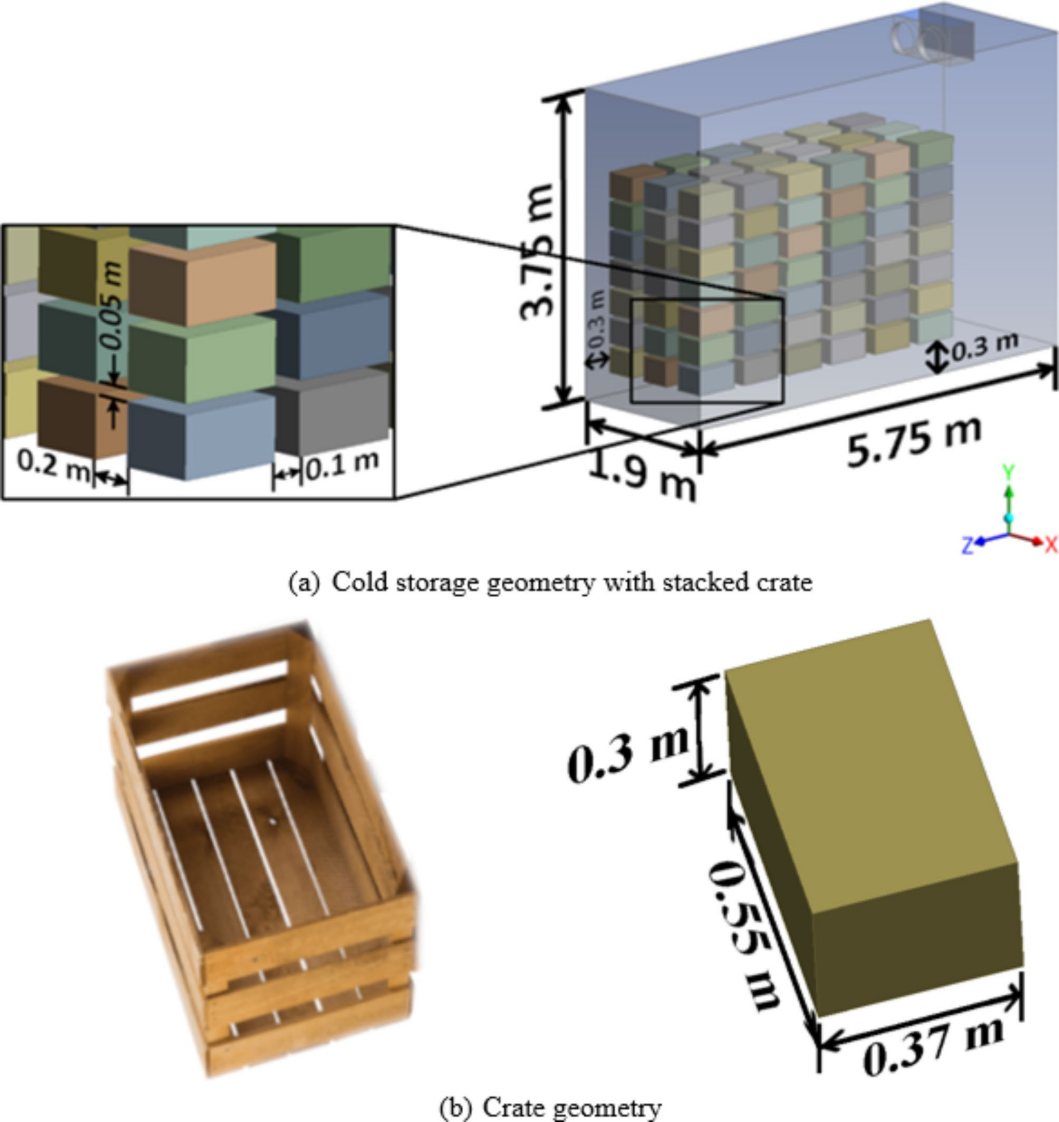
(a) Conventional stacking arrangement (CS1) in cold storage, (b) Crate Dimensions.

Figure 1a depicts the symmetrical half-geometry of cold storage along with the dimensions for which the analysis is carried out. The original cold storage^18^ extends up to 5.75 m in length, 3.8 m in width and 3.75 m in height, with a total void volume of 66.82 m^3^. The cold storage houses 252 number of crates of standard dimension and arranged as shown in Fig. 1b. Since the geometry is symmetric along the YZ plane, only half the geometry is modeled and simulated. Thus, by reducing the width of the geometry by half, i.e., 1.9 m, the number of crates becomes 126, and the total void volume decreases to 33.41 m^3^. The IDU (indoor unit) is suspended from the ceiling at 0.4 m from the rear wall and has two fans each with a diameter of 0.4 m. The cold air is blown at a velocity of 4.2 m s^− 1^ and is recirculated back through the rear side of the IDU. The bottom layer of crates is supported over wooden pallets 0.3 m in height and forms an important air return path to the IDU. The crates are stacked directly over one another in the reported experimental study^18^ leaving only random gaps due to undulations in the construction of wooden crates and stacking errors.

Each crate measures 0.55 m in length, 0.37 m in width, and 0.3 m in height, resulting in a volume of 0.06105 m³. These dimensions are designed to accommodate approximately 20 kg of apples, aligning with the standards of commercially available crates as shown in Fig. 1b^19^. The geometry of the crate along with that of the stored apples are considered to exhibit properties akin to those of porous media, while the details of the interaction of air flow with the stored apples are not studied in this research. Thus, the porous media include the crate, the apples in it and the packaging material, while a restricted amount of air flows through them. The crates are modeled in such a way that they are stacked on top of each other with a specified air gap supported by a racking system. The spacings along the X and Z directions, as per the experimental setup, are taken as 0.2 m and 0.1 m, respectively, while a small uniform air spacing of 0.05 m is modeled along the Y direction to ensure that the columns of crates in a stack are not modeled as monolithic blocks but rather as separated porous blocks.

### Assumptions

- Forced convection is the predominant mode of heat transfer.
- The apple crates are assumed to constitute a single homogenous porous medium.
- The thermophysical properties of apples do not change with temperature.
- The inlet air is assumed to flow normally to the surface of the IDU at a constant velocity.
- The heat capacities of wooden pallets, metallic racks and crates and their effects on the fluid flow are neglected.

### Governing equations

A multiscale simulation is adopted, as it overcomes the requirement for a higher computational load, as in a monoscale simulation. This is achieved by integrating the simulation results obtained from a small domain into the full scale model, thereby significantly reducing the computational cost^20^. The Reynolds average Navier Stokes or RANS equation comprises three dimensional continuity and the momentum equation, which are used to solve the flow field as well as the transient heat transfer inside cold storage^21,22^ as shown in Eqs. (1) and (2).

1 $$\:\frac{\partial\:{\rho\:}_{a}}{\partial\:t}+\stackrel{-}{\nabla\:}\left({\rho\:}_{a}\stackrel{-}{u}\right)=0$$

2 $$\:\frac{\partial\:{\rho\:}_{a}{\stackrel{-}{u}}_{i}}{\partial\:t}+\frac{\partial\:{\rho\:}_{a}{\stackrel{-}{u}}_{i}{\stackrel{-}{u}}_{j}}{\partial\:{x}_{j}}=-\frac{\partial\:\stackrel{-}{p}}{\partial\:{x}_{i}}+\frac{\partial\:}{\partial\:{x}_{j}}\left[\mu\:\left(\frac{\partial\:{\stackrel{-}{u}}_{i}}{\partial\:{x}_{j}}+\frac{\partial\:{\stackrel{-}{u}}_{j}}{\partial\:{x}_{i}}\right)\right]-\frac{\partial\:{\rho\:}_{a}\stackrel{-}{{u}_{i}^{{\prime\:}}{u}_{j}^{{\prime\:}}}}{\partial\:{x}_{j}}+{S}_{i}$$

$\:SST\:k-\omega\:$ turbulence model is used in the simulation to predict the turbulence flow behavior inside cold storage, as recommended^11^. It consists of two transport equations, the turbulent kinetic energy ($\:k$) and the specific turbulent dissipation rate ($\:\omega\:$), which are solved to simulate the turbulence behavior near the plane surface. The momentum equation depicted in Eq. (2) includes the Reynolds stress term, which is simplified according to the Boussinesq hypothesis^23^ as shown in Eqs. (3),

3 $$\:{\rho\:}_{a}\stackrel{-}{{u}_{i}^{{\prime\:}}{u}_{j}^{{\prime\:}}}=-{\mu\:}_{t}\left(\frac{\partial\:{\stackrel{-}{u}}_{i}}{\partial\:{x}_{j}}+\frac{\partial\:{\stackrel{-}{u}}_{j}}{\partial\:{x}_{i}}\right)+\frac{2}{3}{\rho\:}_{a}k{\delta\:}_{ij}$$

The $\:SST\:k-\omega\:$ turbulence model uses a blending function that combines the use of the $\:k-\omega\:$ model for the near-wall region and the $\:k-\epsilon\:$ model for the far field region^24^. The expressions for the turbulent viscosity $\:\left({\mu\:}_{t}\right)$ in the $\:k-\omega\:$ and $\:k-\epsilon\:$ models are shown in (4) and (5),

4 $$\:{\mu\:}_{t}={\rho\:}_{a}\frac{k}{\omega\:}$$

5 $$\:{\mu\:}_{t}={\rho\:}_{a}{C}_{\mu\:}\frac{{k}^{2}}{\epsilon\:}$$

The effect of the porous media on the momentum equation along the cells of the crates is computed by the Darcy-Forchheimer Eq. 2^25^ as shown in Eq. (6). The Darcy-Forchheimer equation is translated into a supplementary source term and added to the momentum Eq. 

6 $$\:{S}_{i}=-\left(\frac{\mu\:}{{C}_{1}}{u}_{i}+\frac{1}{2}{C}_{2}{\rho\:}_{a}\left|u\right|{u}_{i}\right)$$

The former term on the right-hand side of the equation represents the viscous resistance and the latter term represents the inertial resistance of the crates assumed to be porous media. Table 1 shows the viscous and inertial resistance values of apples loaded in crates used for modeling porous media.

**Table 1:** Viscous and Inertial resistance to airflow through apple crates^26^.

Air flow direction	Viscous resistance/m^− 2^	Inertial resistance/m^− 1^
X	3.28 × 10^5^	409.2
Y	1.19 × 10^5^	416.9
Z	3.06 × 10^5^	474.2

The RANS equation for the conservation of energy^27^ is given by Eq. (7)

7 $$\:\frac{\partial\:{\stackrel{-}{T}}_{a}}{\partial\:t}+{\stackrel{-}{u}}_{i}\left(\frac{\partial\:{\stackrel{-}{T}}_{a}}{\partial\:{x}_{i}}+\frac{1}{{\rho\:}_{a}{C}_{pa}}\frac{\partial\:\stackrel{-}{p}}{\partial\:{x}_{i}}\right)=\frac{\partial\:}{\partial\:{x}_{i}}\left(\frac{{k}_{eff}}{{\rho\:}_{a}{C}_{pa}}\frac{\partial\:{\stackrel{-}{T}}_{a}}{\partial\:{x}_{i}}\right)$$

Equation (7) is modified assuming local thermal equilibrium due to the presence of porous media considering that the heat transfers through the solid (apple) and the fluid (air gap) are given by Eq. (8).

8 $$\:\left[\gamma\:{\rho\:}_{a}{C}_{pa}+\left(1-\gamma\:\right){\rho\:}_{s}{C}_{ps}\right]\frac{\partial\:{\stackrel{-}{T}}_{a}}{\partial\:t}+{\rho\:}_{a}{C}_{pa}{\stackrel{-}{u}}_{i}\left(\frac{\partial\:{\stackrel{-}{T}}_{a}}{\partial\:{x}_{i}}+\frac{1}{{\rho\:}_{a}{C}_{pa}}\frac{\partial\:\stackrel{-}{p}}{\partial\:{x}_{i}}\right)=\frac{\partial\:}{\partial\:{x}_{i}}\left[\frac{\partial\:{\stackrel{-}{T}}_{a}}{\partial\:{x}_{i}}\left(\gamma\:{k}_{eff}+\left(1-\gamma\:\right){k}_{s}\right)\right]+{S}_{resp}$$

Equation (8) can be simplified by writing the heat transfer for the solid and fluid zones separately as expressed in Eqs. (9) and (10). The effective thermal conductivity $\:{k}_{eff}$ is calculated using Eq. (11).

9 $$\:\gamma\:\frac{\partial\:{\stackrel{-}{T}}_{a}}{\partial\:t}+{\stackrel{-}{u}}_{i}\left(\frac{\partial\:{\stackrel{-}{T}}_{a}}{\partial\:{x}_{i}}+\frac{1}{{\rho\:}_{a}{C}_{pa}}\frac{\partial\:\stackrel{-}{p}}{\partial\:{x}_{i}}\right)=\gamma\:\frac{\partial\:}{\partial\:{x}_{i}}\left[\frac{\partial\:{\stackrel{-}{T}}_{a}}{\partial\:{x}_{i}}\frac{{k}_{eff}}{{\rho\:}_{a}{C}_{pa}}\right]+{S}_{int}$$

10 $$\:\left(1-\gamma\:\right)\frac{\partial\:{\stackrel{-}{T}}_{s}}{\partial\:t}=\left(1-\gamma\:\right)\frac{\partial\:}{\partial\:{x}_{i}}\left[\frac{\partial\:{\stackrel{-}{T}}_{s}}{\partial\:{x}_{i}}\frac{{k}_{s}}{{\rho\:}_{s}{C}_{ps}}\right]+{S}_{resp}-{S}_{int}$$

11 $$\:{k}_{eff}=\gamma\:{k}_{a}+\left(1-\gamma\:\right){k}_{s}$$

In practice, respiration continues at a retarded rate in harvested products that are stored in cold storage^28^. This implies a continuous chemical reaction in which the fruit and vegetables transform glucose present in them, using oxygen to produce water, carbon dioxide and heat. The culminated respiration heat from the apples is released into cold storage which contributes to the total heat load for the refrigeration system. The respiratory load of the product depends on the temperature and the amount of oxygen in the air in which it is stored. A correlation^29^ is utilized to compute the amount of heat released by the product per unit mass, upon considering the effect of temperature only and is shown in Eq. (12).

12 $$\:{S}_{resp}=\left(1-\gamma\:\right){\rho\:}_{s}\times\:\frac{10.7}{3600}\times\:f{\left[\frac{9{T}_{s}}{5}+32\right]}^{g}$$

The variables $\:f$ and $\:g$ in Eq. (12) correspond to the respiration coefficients of apple, which are 5.6871 × 10^− 4^ and 2.5977, respectively, as reported in the ASHRAE Handbook for Refrigeration^29^. The parameter $\:{S}_{int}$ is the interface term representing the heat exchange between the apples and the air. The values of various parameters for building the model are tabulated in Table 2.

**Table 2:** Model parameters.

Parameter Apple (packed in crates)	Value	Source
Bulk porosity, $\:\gamma\:$	0.614	^18^
True density, $\:{\rho\:}_{s}$	845.4 $\:kg\:{m}^{-3}$	^29^
Bulk density	326.306 $\:kg\:{m}^{-3}$	Calculated
Heat capacity, $\:{C}_{ps}$	3640 $\:J\:{kg}^{-1}\:{K}^{-1}$	^30^
Thermal conductivity, $\:{k}_{s}$	0.50 $\:W\:{m}^{-1}\:{K}^{-1}$	^30^
Air		
Density, $\:{\rho\:}_{a}$	1.28 $\:kg\:{m}^{-3}$	^31^
Heat capacity, $\:{C}_{pa}$	1006.4 $\:J\:{kg}^{-1}\:{K}^{-1}$	^31^
Thermal conductivity, $\:{k}_{a}$	0.02397 $\:W\:{m}^{-1}\:{K}^{-1}$	^31^
Viscosity, $\:\mu\:$	1.72 × 10^–5^$\:N\:s\:{m}^{-2}$	^31^

### Initial and boundary conditions

The entire geometry of the model includes air as the fluid, apple crates as the porous media and cold storage walls that have an initial temperature of 28 °C and are in thermal equilibrium with each other. The boundary conditions are assumed to be the same as those in the experimental study^18^. A symmetric boundary condition is applied on the symmetry wall, which imposes no fluid flow and no scalar flux across the wall^32^. The no-slip boundary condition is assumed on the walls of the IDU, outer surface of the crates, floor, ceiling, and walls of the cold storage, which assumes that the velocity of the air in the immediate vicinity of the wall is zero. The cold air from the IDU is considered a velocity inlet boundary condition with circulating air flowing at 4.2 m s^− 1^ at a constant temperature of 1.4 °C, while the outlet is considered a pressure outlet. To simulate the crates as a porous zone and the circulating air as a single fluid zone, two separate domains were generated. Domain 1 features a porous zone consisting of porous media, while Domain 2 consists of a single fluid material to simulate the circulating air throughout the cold storage bounded by the room walls, the floor, the ceiling, and the symmetry wall.

**Fig. 2:**
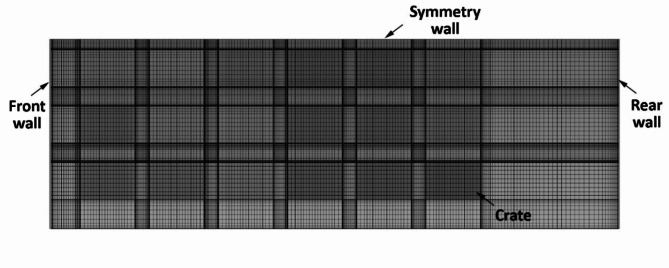
Top view of the generated mesh for the cold storage model.

### Simulation procedure

The simulation is carried out using ANSYS Fluent v18.1, which uses a finite volume approach for solving the partial differential equations associated with the RANS equation. The software was executed on a desktop computer with an Intel Xeon Gold 6230R CPU @ 2.10 GHz and 128 GB of usable RAM. The model was solved with a pressure based transient solver considering the effect of gravity. The SIMPLE (Semi-Implicit Method for Pressure-Linked Equations) algorithm was implemented for the pressure-velocity coupling scheme. Second order upwind differencing was selected for spatial discretization of the momentum, turbulence, and energy equations. The convergence criteria were determined by the normalized root mean square residual error with target values less than 10^− 3^ for the continuity, velocity, $\:k$ and $\:\omega\:$ equations and 10^− 6^ for the energy equation. The transient simulation started at a very small time-step and gradually increased up to 10 s. Figure 2 shows the top sectional view of the mesh generated for cold storage in a section where finer mesh near the crate wall and cold storage walls is visible. The mesh design aimed to accurately capture boundary layer formations with the $\:SST\:k-\omega\:$ turbulence model. This was achieved by ensuring y + values were maintained below 1 throughout near-wall regions, utilizing a structured mesh approach with a growth ratio of 1.2.

A grid independence study or GIS is carried out first to ascertain that the minimum number of mesh elements generated for simulation is sufficient for the geometrical model and that a further increase in the mesh density introduces no observable improvement. The Richardson extrapolation method^33^ is selected for the GIS, as it has been successfully utilized in the simulation of cold storage^22,34^. Hexa-dominant mesh elements were used for the discretization of the computational domain, the geometrical model of cold storage considered for the GIS. Six different numbers of mesh elements were taken with a grid refinement ratio in the range of 1.4–1.5 to ensure that the selection of grid spacing did not fall outside the asymptotic range of convergence. The grid independence test was conducted for a range of average grid spacings ranging from 0.125 m (coarsest mesh with 2 × 10^4^ elements) to 0.02 m (finest mesh with 5 × 10^6^ elements).

**Fig. 3:**
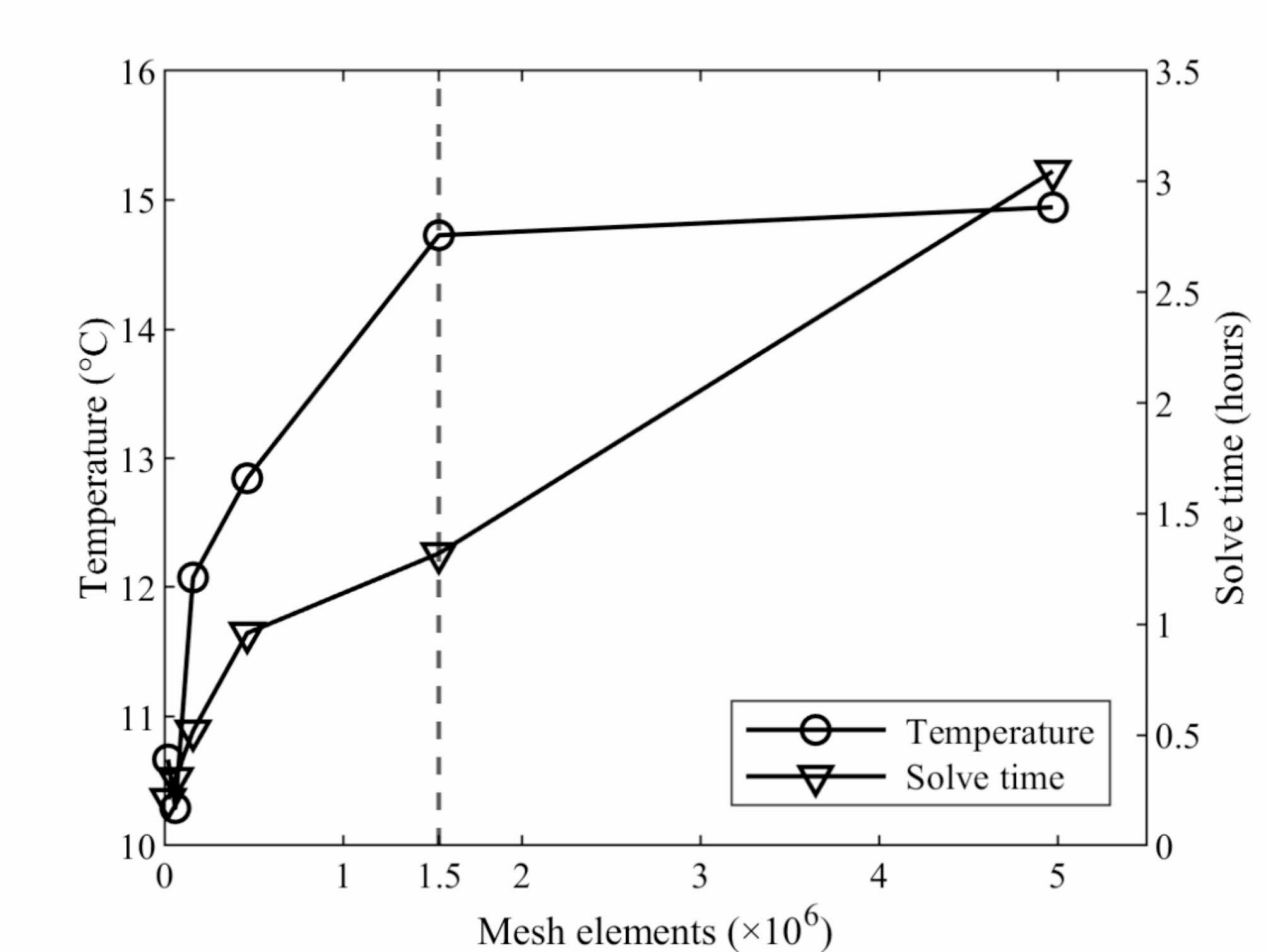
Grid Independence study.

Figure 3 shows the GIS results for 1800 s of flow time with the number of mesh elements provided on the horizontal axis, the temperature on the primary vertical axis and the solve time on the secondary vertical axis. Here, solve-time represents the computation time required to simulate the cold storage model for the given conditions. The optimum grid spacing was determined to be 1.5 × 10^6^ mesh elements since a further increase in mesh density did not affect the result while achieving similar outcome at 56.7% lesser solve time than its successor (5 × 10^6^ elements). The average discretization error corresponding to the selected number of mesh elements is found to be 1.56%, which is in line with previous literature^22,34^.

### Model validation

**Fig. 4:**
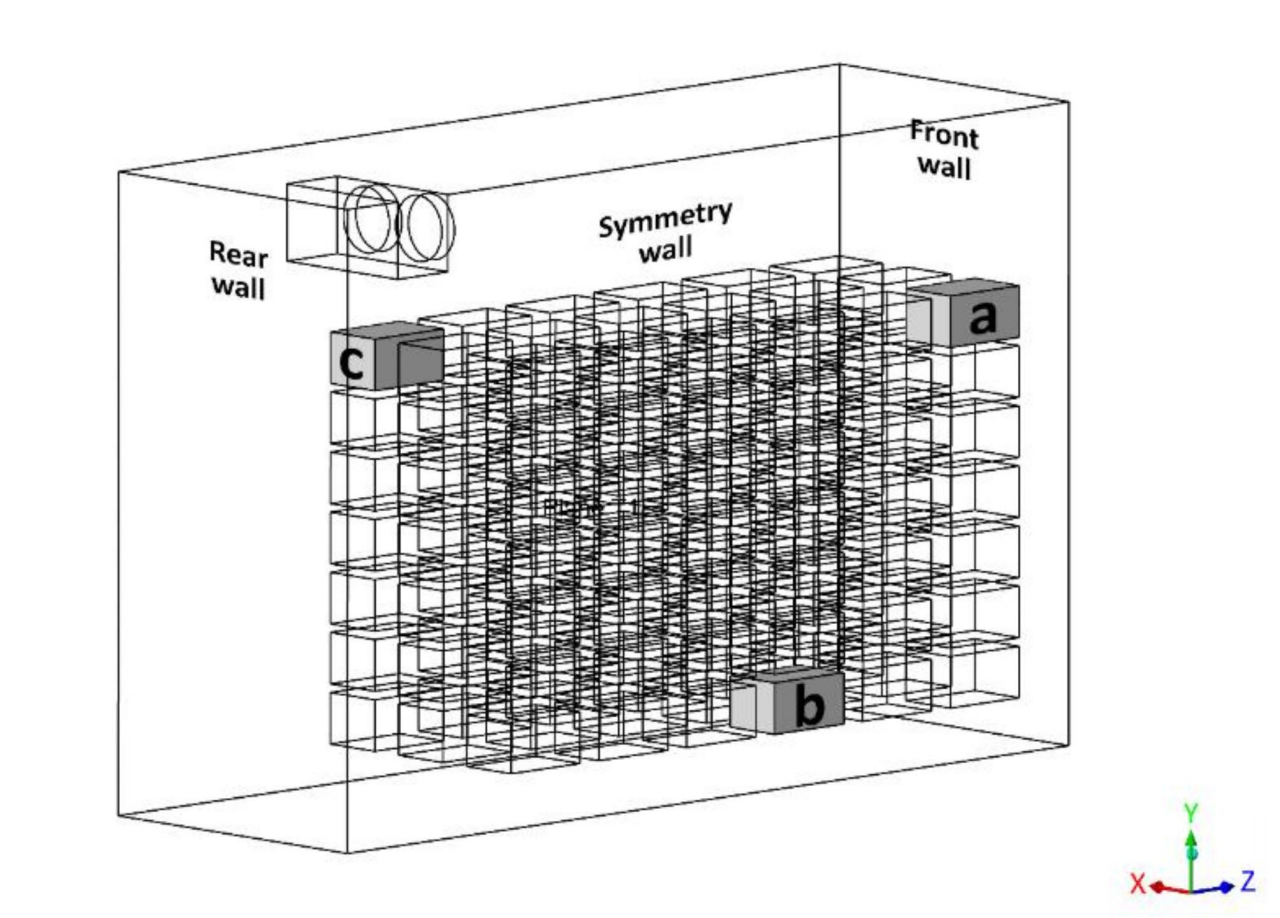
Location of crates a, b, and c.

**Fig. 5:**
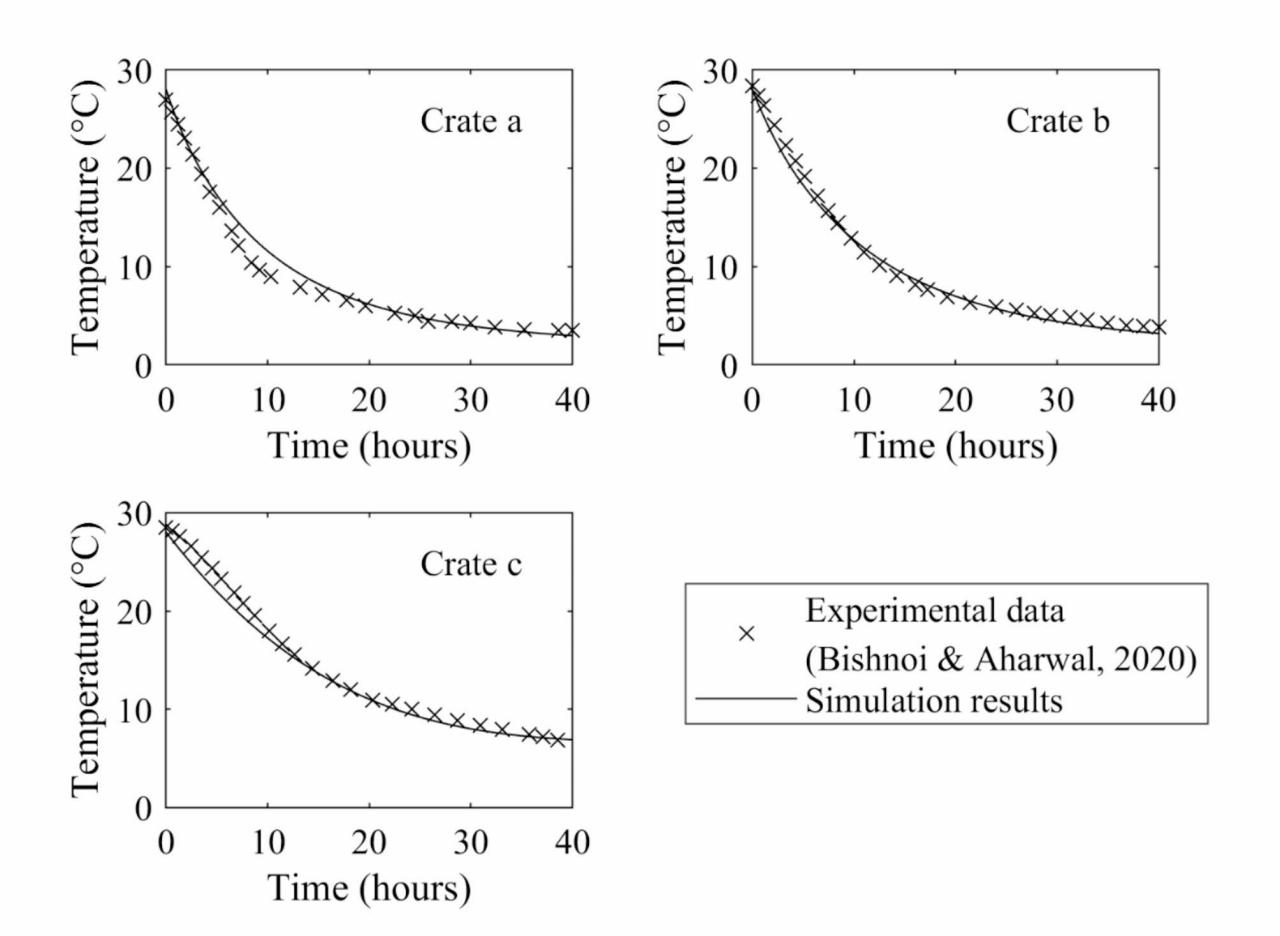
Comparison of simulation results with experimental data for crate a, crate b, and crate c inside cold storage with the CS1 configuration.

Figure 4 indicates the selected positions of the crates for which the temperature data were recorded. These positions (a, b and c) were strategically selected to study the effect of the air flow distribution on the temperature gradient of crates near the front wall, rear wall, and floor of the cold storage. Since the focus of this study is on the ways in which crates cool, it was considered necessary and sufficient to validate the results against published temperature data. Figure 5 summarizes the comparison between the experimental data and simulated results for crates at these three positions. Both experimental data and CFD results show that the crates cool asymptotically by exchanging heat with the surrounding chilled air, consistent with observations in previous literature^35,36^. The calculated root mean square errors for the crates position a, b and c are found to be 1.13 °C, 0.71 °C and 0.817 °C which indicates a good fit. Thus, the numerical model is considered validated. The comparison between the experimental data and the CFD results of the parametric study can be found in the Supplementary Data.

### Simulation for various crate orientations and spacings

**Fig. 6:**
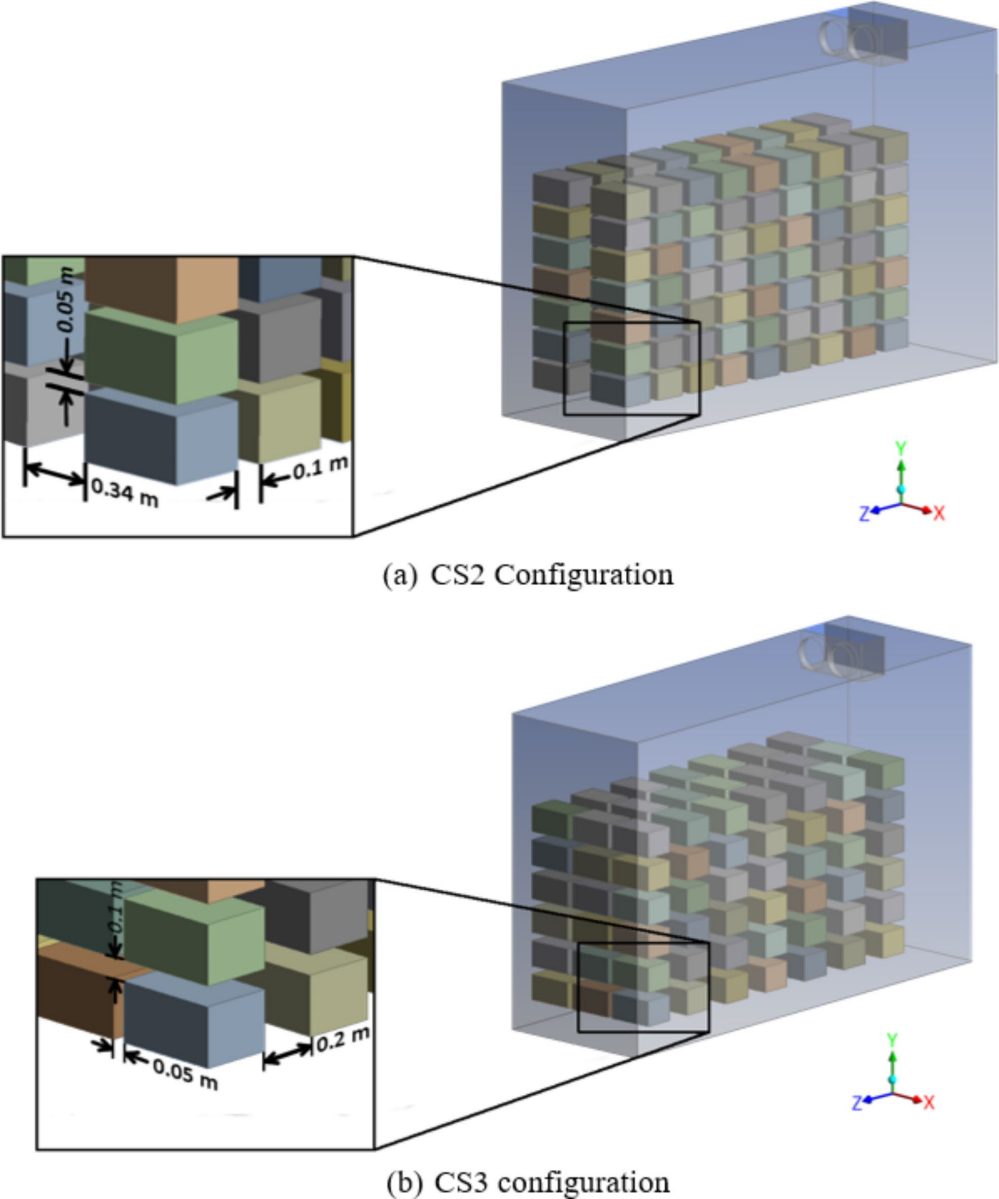
Proposed stacking arrangement of crates in cold storage (a) CS2 stacking arrangement and (b) CS3 stacking arrangement.

The uneven cooling of the crates observed in the CS1 configuration during cold storage may be attributed to the modification of the stacking configuration of the crates. There are two main parameters that affect the cooling rate achieved inside cold storage: the orientation at which the crates are stored relative to the air flow from the IDU and the space between two adjacent crates along the X, Y and Z directions. To study the effect of these parameters, two different stacking configurations, CS2 and CS3, were simulated, as shown graphically in Fig. 6. The motivation behind arriving at these two configurations is to study the cooling effect of the crates in different orientations and how the cooling rate is affected by increasing the spacing along the y direction while maintaining the same total number of crates in the storage space constant as well as the minimum distance of crates from the boundary walls.

**Fig. 7:**
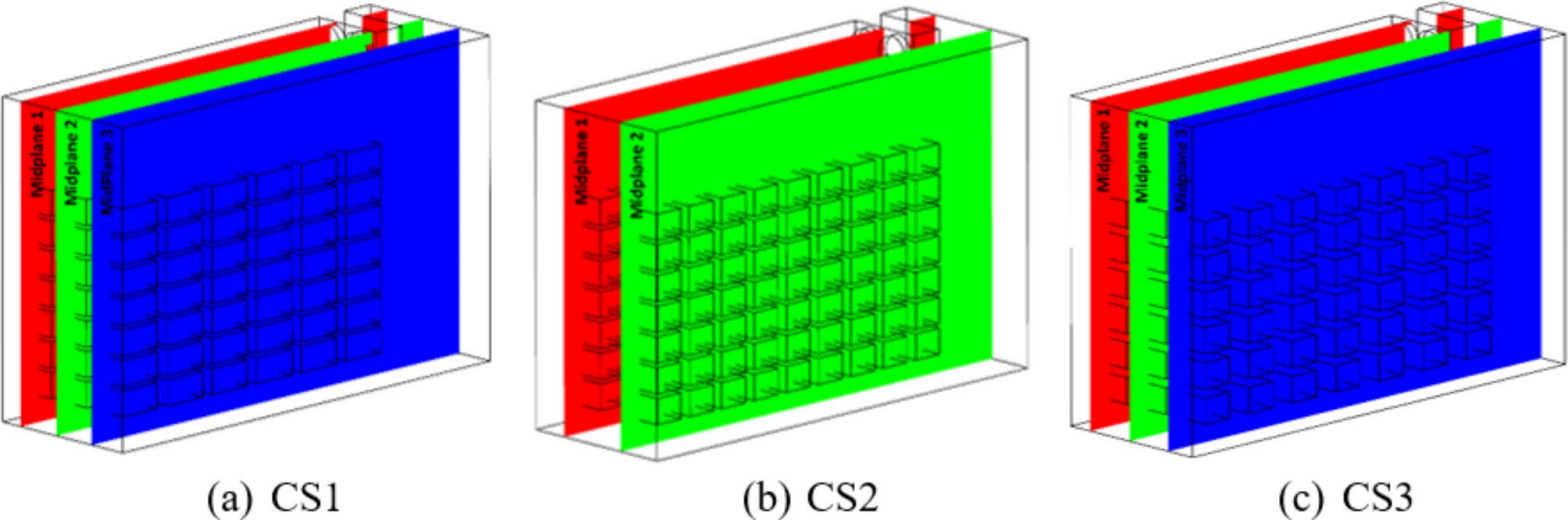
Reference midplanes located at the middle of each column in the CS1 (**a**), CS2 (**b**) and CS3 (**c**) stacking configurations.

Note that the parameters pertaining to configuration CS1, such as the dimensions of the crate and cold storage, the void volume, and the total number of crates in the cold storage, inlet and outlet cold air conditions, were the same while simulating CS2 and CS3 configurations. In the CS2 and CS3 configuration, the orientation of the crates has been changed to study its influence on the crate temperature. Table 3 provides a comprehensive overview of the modifications made to the proposed CS2 and CS3 configurations. As a result of the change in intermediate crate spacing in the CS2 and CS3 configurations, the number of crates placed along the three directions changes, without changing the total number of crates. To examine the temperature in the crates at different locations inside the cold storage, cut section planes in the middle of the crates (midplane 1, midplane 2 and midplane 3) along the Z-axis in all three stacking configurations are depicted in Fig. 7.

**Table 3:** Proposed alternative stacking configurations.

	Orientation	Change in intermediate spacing (%)		
		X direction	Y direction	Z direction
CS2 configuration	Opposite to CS1	70% increase	No change	No change
CS3 configuration	Opposite to CS1	75% reduction	100% increase	100% increase

### Temperature heterogeneity

Temperature heterogeneity^37^ is the non-uniformity of the product temperature at various locations inside cold storage. The temperature heterogeneity^17^ is computed using the dimensionless temperature parameter $\:{Y}_{i}$ (Eq. (13)).

13 $$\:{Y}_{i}=\frac{{T}_{s,t}-{T}_{a}}{{T}_{s,in}-{T}_{a}}$$

The temperature heterogeneity among the crates stacked in three different configurations is calculated using Eq. (14). The equation captures the temperature heterogeneity of the apple crates by computing the difference between the maximum and minimum values of the dimensionless temperature parameter $\:{Y}_{i}$ at each time step $\:t$.

14 $$\:\varDelta\:\:=max\left|{Y}_{i}-\frac{1}{n}\sum\:_{i=1}^{n}{Y}_{i}\right|-min\left|{Y}_{i}-\frac{1}{n}\sum\:_{i=1}^{n}{Y}_{i}\right|$$

## Results and discussion

The subsequent section highlights the results from the numerical model investigation. A detailed compilation of all findings from the parametric study is available in Supplementary Data.

### Velocity distribution

**Fig. 8:**
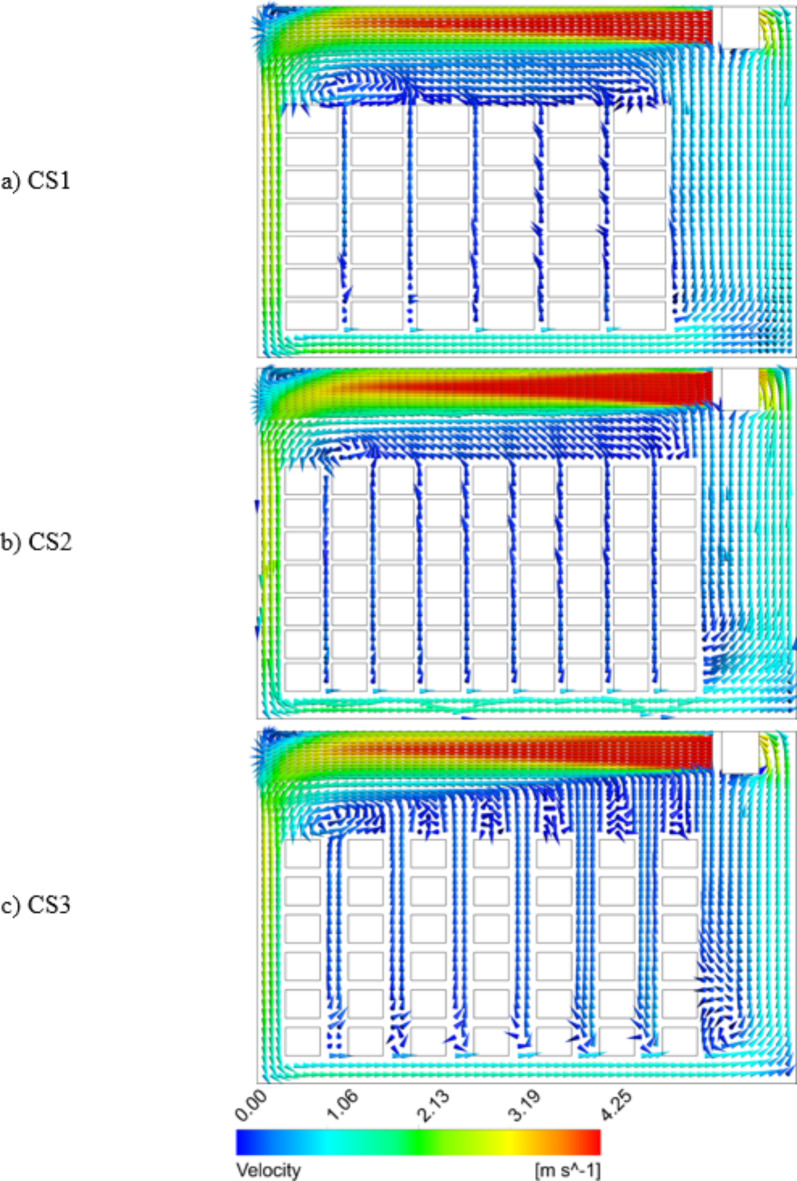
2D vector plot for midplane 1 in the CS1 (**a**), CS2 (**b**) and CS3 (**c**) stacking configurations during cold storage.

Figure 8 depicts 2D vector plot for Midplane 1 in the CS1, CS2 and CS3 configurations. The cold air emanating from the outlet of the IDU flows across the cold storage towards the rear wall at a gradually decreasing speed and then spreads across the crates from the bottom. The cold air disperses into cold storage, as it interferes with the surrounding air and the apple crates. The average y-direction velocities of cold air amidst the column of crates in CS1 and CS2 configurations are computed to be 0.1 m s^− 1^ and 0.05 m s^− 1^ respectively, due to spacing restrictions, while the same velocity is found to be as high as 0.42 m s^− 1^ in the CS3 configuration due to larger spacing.

Figure 8 also shows that a significant amount of air with a higher velocity engages with the crates in the CS3 configuration compared to the CS1 and CS2 configurations. This is apparent in the 2D vector plot, where the predominance of an increased number of upward arrows signifies a greater volume of air movement. A higher amount of air flowing between the crates ensures adequate cooling of the crates and reduces the bypass of cold air through the cold room floor. This bypassed air short circuits back to the IDU and resulted in inefficient cooling. The increase in crate spacing along the Z direction and subsequent reduction along the X direction ensures that the cold air is not discharged back to the IDU and remains in contact with the crates longer.

### Temporal distribution of temperature

**Fig. 9:**
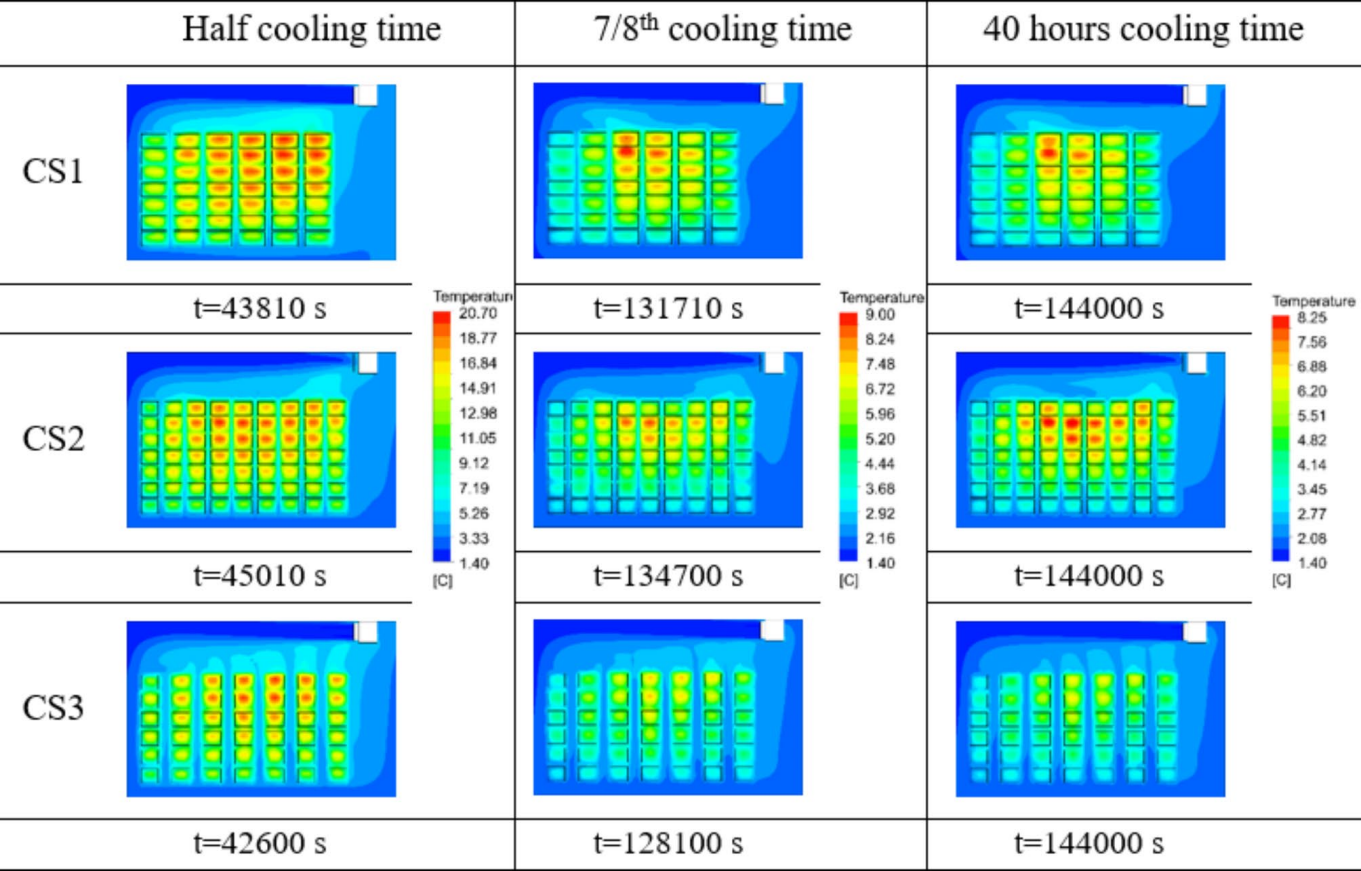
Temperature contours in Midplane 1 for CS1, CS2 and CS3 configurations of cold storage at half cooling time, 7/8th cooling time and at 40th hour cooling time.

The temporal distributions of the temperature in midplane 1 for CS1, CS2 and CS3 are shown in Fig. 9. To ensure that the comparisons among the various stacking configurations are relevant, the half cooling time and 7/8th cooling time of the crates are considered as reference temporal points^38^. The half cooling time is the time required to reduce the temperature gradient between the stored commodity and cooling medium by half of the initial value. The 7/8th cooling time refers to the time required to remove seven-eighths (87.5%) of the temperature difference between the commodity temperature and the cooling medium. The equations related to calculating both the parameters are given in Eqs. (15) to (17). The maximum crate temperatures in the CS3 configuration at half cooling time, 7/8th cooling time and 40 h of cooling time are 21.2 °C, 8.61 °C and 7.44 °C respectively. Comparatively, the crates stored in the CS3 configuration are 10%, 24% and 25.2% cooler than those stored in the other configurations at their respective times.

15 $$\:{\tau\:}_{\raisebox{1ex}{$1$}\!\left/\:\!\raisebox{-1ex}{$2$}\right.}=\frac{\text{ln}2}{CR}$$

16 $$\:{\tau\:}_{\raisebox{1ex}{$7$}\!\left/\:\!\raisebox{-1ex}{$8$}\right.}=\frac{\text{ln}8}{CR}$$

17 $$\:CR=\frac{\text{ln}{T}_{s,in}-\text{ln}{T}_{s,fi}}{t}$$

**Fig. 10:**
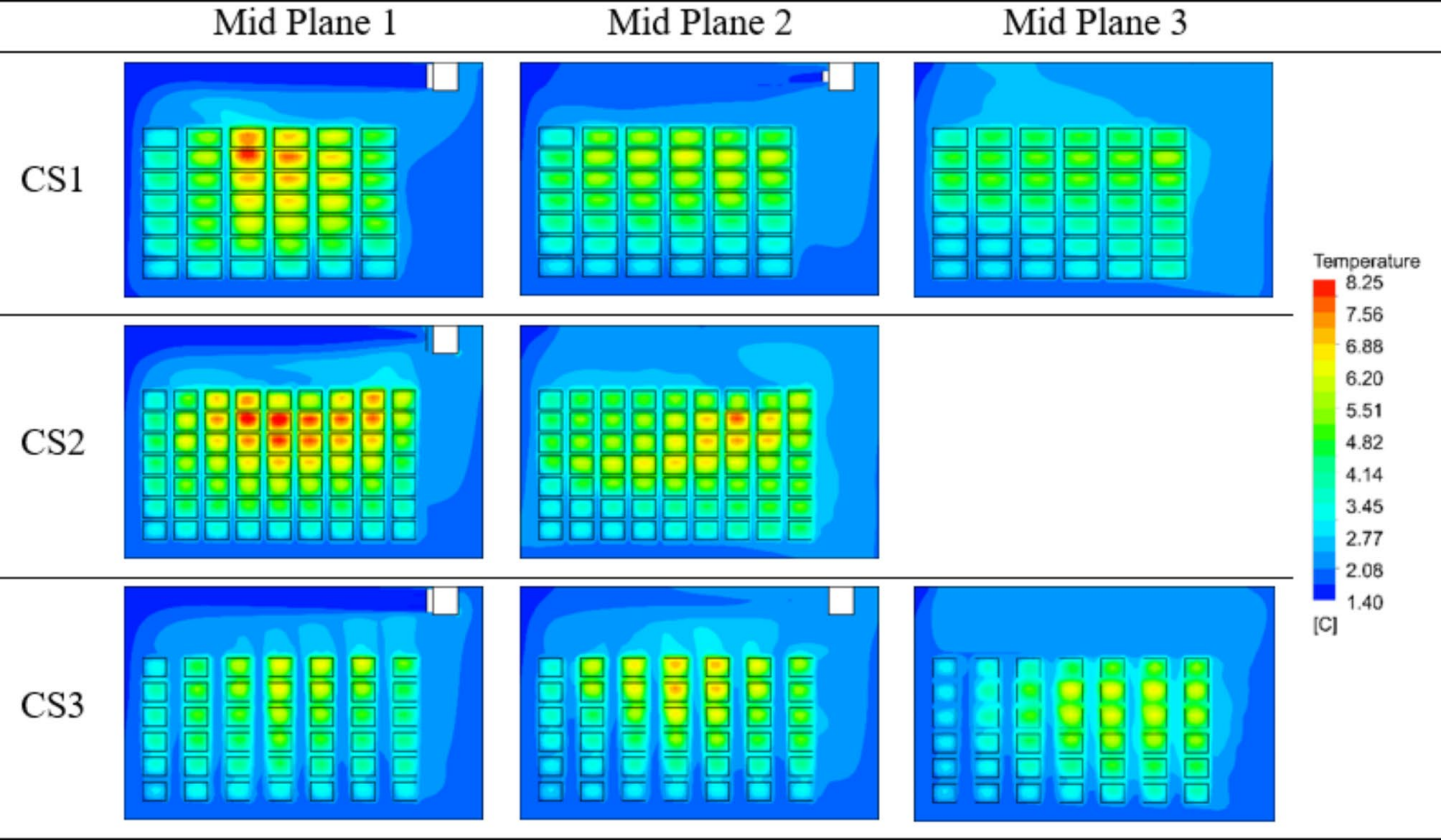
Temperature contours at 40 h flow time at the midplanes for the stacking configurations CS1, CS2 and CS3.

To visualize the temperature heterogeneity of crates inside cold storage at midplanes, the temperature at 40 h of simulation time is shown in Fig. 10. The crates in the middle of the cold storage, which are near the symmetry wall and surrounded by a greater number of crates on all sides, exhibit a higher temperature than the other plane sections in the CS1 and CS2 configurations. This is because the circulating air flow does not reach to the crates located in the middle. This phenomenon is less prominent in CS3. Maximum temperatures of 8.2 °C, 5.92 °C and 6.59 °C are observed in midplane 1, midplane 2 and midplane 3 of the CS1 configuration, respectively. In the CS2 configuration, maximum temperature of 8.14 °C is obtained for midplane 1, while midplane 2 records a maximum of 7.38 °C. The CS3 configuration has the lowest maximum crate temperature compared to the other configurations, which is 7.44 °C in midplane 2, 6.7 °C in midplane 1 and 6.69 °C in midplane 2 respectively. Comparing CS1 (conventional stacking) and CS3 (proposed stacking), the temperature difference achieved in crates arranged in CS3 configuration is 18.63% lower in midplane 1 and 1.52% higher in midplane 3. Likewise, comparison of CS2 and CS3 shows that the maximum crate temperature in CS3 is 17.7% lower in midplane 1, whereas it is 0.8% higher in midplane 2.

### Temperature heterogeneity and histogram data

**Fig. 11:**
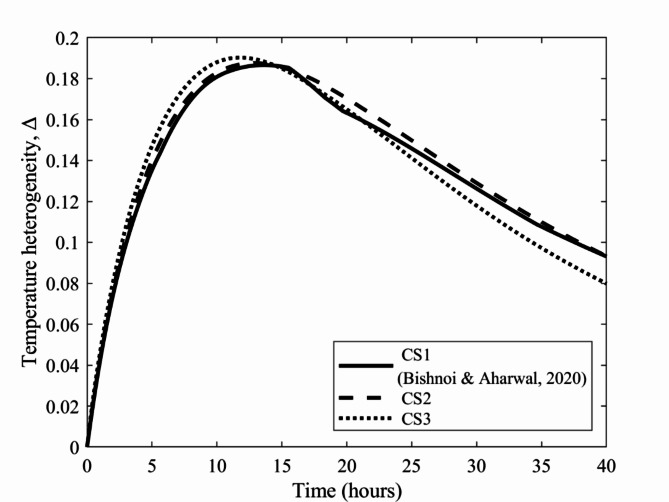
Temperature heterogeneity of crates in cold storage with the CS1, CS2 and CS3 configurations.

Figure 11 shows the temperature heterogeneity for the various stacking configurations CS1, CS2 and CS3. The maximum temperature heterogeneity for the CS1 stacking configuration is 0.18 at 13.67 h. Similarly, for the CS2 configuration, the temperature is 0.18 at 12.83 h, while for the CS3 stacking configuration, the maximum temperature heterogeneity reaches 0.19 at 11.75 h. The temperature heterogeneity of the crates arranged in the CS3 configuration increases rapidly in the early stage due to the higher air flow at the bottom of the cold storage, resulting in a larger temperature gradient than that of the crates at the top. Eventually, after 40 h of storage, the crates in the CS3 configuration achieved the lowest temperature heterogeneity, approximately 14.52% lower than those in the CS1 and CS2 configurations. This stems from the spacing of the crates in CS3, which paves the way for a higher air flow velocity, resulting in a lower temperature gradient (faster cooling) among the crates.

**Fig. 12:**
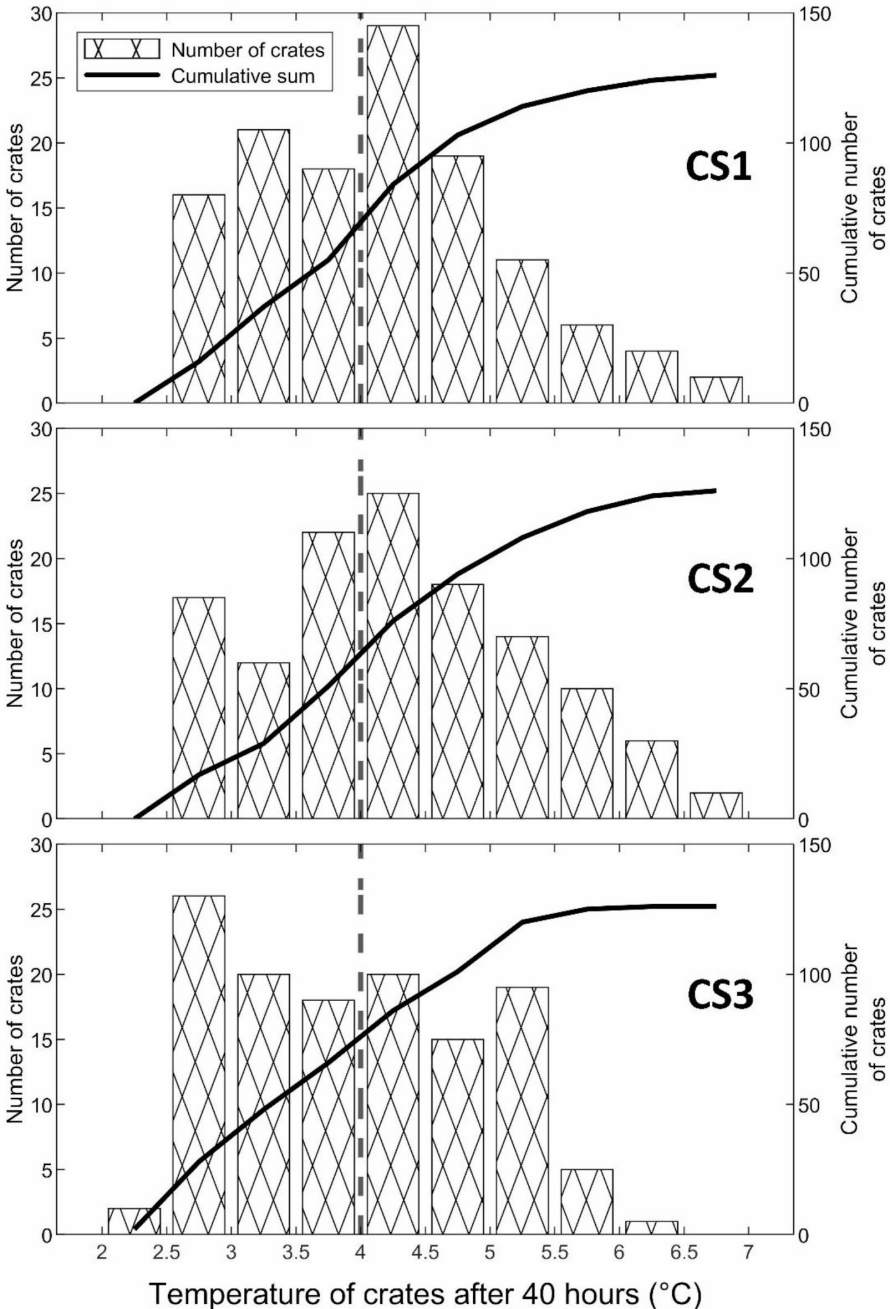
Temperature histogram data for the CS1, CS2 and CS3 configurations.

As previously discussed, for long term storage of apples inside cold storage, it is crucial for the holding temperature to be maintained below 4 °C^39^ with the aim of reducing the degradation of apples due to microbial activity. Figure 12 shows the temperature histogram data for the three stacking configurations at 40 h of cooling. To distinguish between the number of crates that achieved the required storage temperature and the remaining crates, a dotted line is shown at 4 °C. In the CS3 configuration, the number of crates that are below the 4 °C mark is 66, while in the CS1 and CS2 configurations, the same condition prevails only for 55 and 51 crates. The number of crates cooled in the CS3 configuration is 20% greater than that in the CS1 configuration and 29.4% greater than that in the CS2 configuration. It can also be inferred from Fig. 12 that the CS3 configuration does not consist of any crates above 6.5 °C after 40 h, which is not achieved for other configurations. Figure 12 also depicts the cumulative number of crates that are cooled between 2 °C and 7 °C. It can be deduced from the figure that the gradient with which the crates are cooled in the CS3 configuration is larger than that in the CS1 and CS2 configurations. This implies that the crates in the CS3 configuration cooled at a faster rate, thus highlighting the advantage that CS3 is a better arrangement than the other stacking configurations that were examined.

## Conclusion

This research presents the development, meshing, and simulation of a 3D numerical model for an apple cold storage facility that is validated based on published experimental results. This study examines three different stacking configurations using a multiscale modeling approach. The apple-filled crates were modeled as a porous medium. This modeling technique was used to reduce the computational load of simulating the flow dynamics around a real apple in a container. This study analyzed three different stacking orientations, termed CS1, CS2, and CS3, each distinguished by different air gaps between crates. The goal was to study how various arrangements of stacked crates affect airflow dispersion and its influence on temperature distribution over time and location. The major outcomes are follows:

- Validated grid independent model for a conventional (CS1) stacking setup using published data comparing temperature data of three crates from separate locations. The RMSE values of 1.13, 0.71, and 0.817 °C correlated well with the experimental and simulation results.
- Alternate stacking configurations (CS2 and CS3) were designed and simulated with varying orientations and intermediate crate spacings. The average cold air velocity at intermediate of crate spacings computed was 0.1 m s^–1^, 0.05 m s^–1^, and 0.42 m s^–1^ for the CS1, CS2, and CS3 stacking configurations, respectively.
- The CS3 configuration showed crate temperatures that were by 10%, 24% and 25.2% lower than those of conventional CS1 configuration at half, 7/8th, and 40 h, respectively.
- Crate temperature analysis was conducted for three different crate arrangements in cold storage setups at three parallel planes. The maximum crate temperature in the CS3 configuration was 18.63% lower in plane 1, 25.67% higher in plane 2, and 1.52% higher in plane 3 than that in CS1.
- Compared with the other configurations, the CS3 configuration had the lowest temperature heterogeneity after 40 h of cooling. The CS3 stacking of crates had 14.1% and 14.5% lower temperature heterogeneities than those of CS1 and CS2, respectively. In symmetric half of the cold storage tested, in the CS3 configuration, a total of 66 crates achieved 4 °C or lower, which is critical for long-term apple preservation, while in the CS1 and CS2 configurations, the same trend was achieved for 16.7% and 22.7% fewer crates, respectively.

This research shows that parameters such as the crate orientation and intermediate crate spacing have profound effects on the air flow distribution, crate temperature and temperature heterogeneity inside a cold storage. This study also provides further insight into the progress of the cooling process in cold storage. In future work, an optimum stacking configuration with variable air gaps across cold storage can be investigated, which may be achievable in practice with an increasing level of automation in cold storage handling.

## Electronic supplementary material

Below is the link to the electronic supplementary material.

Supplementary Material 1

## Data Availability

Data is provided within the manuscript or supplementary information files.

## List of symbols

$C_{1}$: Darcy permeability (Viscous resistance) $\left( {m^{ - 2} } \right)$
$C_{2}$: Forchheimer drag coefficient $\left( {m^{ - 1} } \right)$
$$C_{p}$$: Sensible heat $$\left( {J kg^{ - 1} K^{ - 1} } \right)$$
$C_{\mu }$: Turbulence model constant
$$CR$$: Cooling rate $$\left( {K s^{ - 1} } \right)$$
$g$: Acceleration due to gravity $\left( {m s^{ - 2} } \right)$
$$I$$: Turbulence intensity ($$\%$$)
$k$: Thermal conductivity $\left( {W m^{ - 1} K^{ - 1} } \right)$/Turbulent kinetic energy $\left( {J kg^{ - 1} } \right)$
$l$: Turbulent length scale $\left( m \right)$
$$p$$: Pressure $$\left( {N m^{ - 2} } \right)$$
$$S$$: Source term
$$Sc$$: Schmidt number
$$t$$: Time $$\left( s \right)$$
$$T$$: Temperature $$\left( K \right)$$
$$u,U$$: Velocity $$\left( {m s^{ - 1} } \right)$$
x: Displacement $$\left( m \right)$$

## Greek

$$\gamma$$: Porosity
$$\delta_{ij}$$: Kronecker delta
$$\mu$$: Dynamic viscosity $$\left( {N s m^{ - 2} } \right)$$
$$\mu_{t}$$: Turbulent viscosity $$\left( {N s m^{ - 2} } \right)$$
$$\rho$$: Density $$\left( {kg m^{ - 3} } \right)$$
$\omega$: Specific turbulent dissipation rate $\left( {s^{ - 1} } \right)$
$$\tau$$: Cooling time $$\left( s \right)$$

## Sub-script

a: Air
eff: Effective values
e: Energy parameter
fi: Final
i: Vector representing x direction
in: Initial
int: Heat exchange between the apple and the air
j: Vector representing y direction
resp: Respiration heat
s: Solid (apple)
w: Water vapor
